# Which spatiotemporal parameters are predicted by commonly used osteoarthritis questionnaires in clinical practice? An exploratory study

**DOI:** 10.1186/s12891-026-09812-y

**Published:** 2026-04-18

**Authors:** Gulnihal Deniz, Omer Esmez, Gokhan Alkan, Furkan Bilek

**Affiliations:** 1 Department of Physiotherapy and Rehabilitation, Faculty of Health Sciences, Erzurum Technical University, Erzurum, 25055 Türkiye; 2 Department of Orthopedics, Fethi Sekin City Hospital, Elazig, Türkiye; 3 Division of Rheumatology, Department of Physical Medicine and Rehabilitation, Faculty of Medicine, Firat University, Elazig, Türkiye; 4 Department of Gerontology, Fethiye Faculty of Health Sciences, Mugla Sıtkı Kocman University, Mugla, 48300 Türkiye

**Keywords:** Knee osteoarthritis, Spatiotemporal gait parameters, Total double support, Step length, Gait analysis

## Abstract

**Background:**

Knee osteoarthritis (KOA) leads to altered gait patterns, yet the relationship between objective spatiotemporal gait parameters and subjective clinical assessment tools remains unclear. This study aimed to examined the relationship between spatiotemporal gait parameters and commonly utilized KOA assessment questionnaires in clinical practice.

**Methods:**

A prospective, cross-sectional study was conducted with 30 patients diagnosed with Kellgren-Lawrence Grade 3 bilateral KOA, randomly selected from a university hospital outpatient clinic. Demographic data, including age and body mass index, were recorded. Spatiotemporal gait parameters were assessed using instrumented gait analysis. Disease severity was evaluated using validated self-reported questionnaires: the Lequesne Index, Oxford Knee Score, Pain Catastrophizing Scale (PCS), and WOMAC subscales (Pain, Joint Stiffness, Function, and Total Score). Correlation and regression analyses were performed to identify associations between gait parameters and osteoarthritis severity scores.

**Results:**

Significant correlations were found between gait parameters and osteoarthritis scales. Total double support duration was positively associated with the Lequesne (*r* = 0.511, *p* = 0.009), Oxford (*r*=-0.455, *p* = 0.022), and WOMAC (*r* = 0.448, *p* = 0.025), indicating a link between prolonged double support time and disease severity. Step length was negatively correlated with PCS (*r*=-0.477, *p* = 0.016), suggesting that greater pain catastrophizing was associated with reduced step length. Regression analysis revealed that total double support time was the strongest predictor of the Lequesne (36.4%), Oxford (43.6%), and WOMAC (36.4%), highlighting its clinical significance.

**Conclusion:**

Spatiotemporal gait parameters, particularly total double support time and step length, are closely associated with patient-reported KOA symptom severity. These parameters may serve as objective clinical indicators for monitoring disease progression and informing rehabilitation strategies.

## Introduction

Osteoarthritis (OA) is a degenerative joint disease characterized by progressive cartilage degradation, osteophyte formation, subchondral sclerosis, and structural changes in the synovial membrane and joint capsule [1]. The global burden of OA has increased substantially, rising from 247.51 million cases in 1990 to 527.81 million in 2019, representing a 113.25% increase [2]. Although the exact pathogenesis of OA remains unclear, several individual factors, including lifestyle, medical history, and physical activity, are known to influence its development [3].

The knee joint is among the most commonly affected sites, and knee osteoarthritis (KOA) is a major cause of pain and physical disability in older adults [4]. KOA may involve one or more compartments of the knee joint, including the medial and lateral femorotibial or patellofemoral compartments [5]. Epidemiological studies indicate that symptomatic KOA affects approximately 13% of women and 10% of men aged 60 years and older, with both prevalence and severity generally higher in women [6]. Clinically, KOA is characterized by symptoms such as pain, joint stiffness, reduced range of motion, instability, and functional limitations, which often lead to gait abnormalities, decreased mobility, and impaired quality of life [7, 8]. These symptoms typically develop gradually and progressively over time [9].

Gait analysis provides valuable insights into the functional consequences of KOA by evaluating spatiotemporal parameters such as step length, stride duration, swing duration, and total double support time [10]. These parameters reflect biomechanical adaptations to pain, joint instability, and muscle weakness, making them critical disease severity and progression indicators. Investigating the relationship between questionnaires assessing KOA symptoms and spatiotemporal gait parameters is particularly important due to the limited accessibility and high cost of gait analysis systems. In clinical practice, osteoarthritis-specific questionnaires, including the Lequesne Index, Oxford Knee Score, the Western Ontario and McMaster Universities Osteoarthritis Index (WOMAC), and Pain Catastrophizing Scales (PCS), are widely utilized to assess the impact of KOA on patients’ physical function and pain perception [4, 10]. These tools provide subjective measures of disease burden by capturing patients’ experiences of pain, stiffness, and mobility restrictions. However, while these self-reported questionnaires offer valuable insights into perceived disability and quality of life, they may not fully account for the objective biomechanical impairments associated with KOA [4, 10]. Although these questionnaires effectively assess patient-reported outcomes, their ability to predict specific spatiotemporal gait parameters remains incompletely understood. Given the clinical significance of stride duration, swing duration, and total double support time, further research is needed to elucidate their associations with osteoarthritis-specific assessment scales and their potential role in informing patient-centered interventions [4, 10].

Spatiotemporal gait analysis provides an objective and quantifiable method for evaluating functional limitations by identifying gait abnormalities associated with joint degeneration and pain-induced compensatory movements [4, 10] Obesity, a progressively prevalent comorbidity among individuals with KOA, is recognized for its role in exacerbating disease progression and altering lower extremity biomechanics. Increased body weight imposes excessive mechanical loading particularly in the medial compartment of the knee, which may contribute to cartilage degeneration and altered gait mechanics [11]. This increased loading may prompt adaptive gait strategies such as prolonged double support duration and decreased step length to mitigate joint stress and maintain postural stability.

Obesity is a common comorbidity among individuals with knee osteoarthritis and may influence lower extremity biomechanics and gait characteristics. Increased body weight imposes greater mechanical loading on the knee joint, particularly in the medial compartment, which may contribute to altered gait strategies. In the present study, BMI classification was also examined to explore potential differences in spatiotemporal gait parameters and questionnaire scores among participants. Therefore, the primary aim of this study was to investigate the relationship between spatiotemporal gait parameters and commonly used osteoarthritis questionnaires, with BMI classification considered as a secondary factor that may influence gait characteristics and patient-reported outcomes.

## Material methods

### Study design

This prospective cross-sectional study examined the relationship between spatiotemporal gait parameters and commonly used KOA questionnaires. The study was conducted at the Orthopedics and Traumatology and Physical Medicine and Rehabilitation outpatient clinics of Firat University Hospital from July 2024 to January 2025. Thirty patients with bilateral KOA (Kellgren–Lawrence Grade 3) who met the inclusion criteria were consecutively recruited from patients attending these outpatient clinics during the study period. The collected data included age, body mass index (BMI), osteoarthritis severity scores obtained from patient-reported questionnaires (WOMAC, Oxford Knee Score, and Lequesne Index), and spatiotemporal gait parameters. Gait parameters were assessed via instrumented gait analysis, while KOA severity was evaluated using the Lequesne Index, Oxford Knee Score, PCS, and WOMAC subscales. Surveys took 30 min to complete. The study was ethically approved on June 6, 2024 (protocol 2024/09 − 07) and adhered to the Declaration of Helsinki (Clinical trial number: Not applicable). Written informed consent was obtained.

### Participants

A priori power analysis was conducted using G*Power (version 3.1.9.4) based on a bivariate correlation test to determine the required sample size for detecting associations between spatiotemporal gait parameters and questionnaire scores. The analysis indicated a high effect size (0.68) with a statistical power of 0.95 at a significance level of 0.05 [12]. Based on this analysis, a total of 30 patients with bilateral KOA who had not undergone total knee arthroplasty (TKA) were included in the study. Only patients with bilateral knee osteoarthritis classified as Grade 3 according to the Kellgren–Lawrence grading system were included to ensure a homogeneous sample with comparable disease severity. Grade 3 represents a moderate to advanced stage of osteoarthritis, in which structural joint changes and functional impairments are more pronounced and more likely to be reflected in spatiotemporal gait parameters. Limiting the sample to this radiographic stage minimized heterogeneity and allowed a clearer evaluation of the relationship between gait parameters and clinical assessment questionnaires. Exclusion criteria included individuals with a BMI > 40 kg/m², those enrolled in a physical therapy program, those unable to stand for at least 10 min, individuals requiring assistive devices for ambulation, and those with comorbid conditions affecting lower extremity gait function. Participants with BMI > 40 kg/m² were excluded to minimize the potential confounding effects of morbid obesity on gait mechanics, as severe obesity may independently alter spatiotemporal gait parameters.

### Data collection tools

In this study, the validated Turkish versions of the Lequesne Index, Oxford Knee Score, PCS, and WOMAC were used. These instruments have previously undergone cross-cultural adaptation and validation for use in Turkish populations and are widely used in non-commercial academic research.

#### Spatiotemporal parameters

**Fig. 1:**
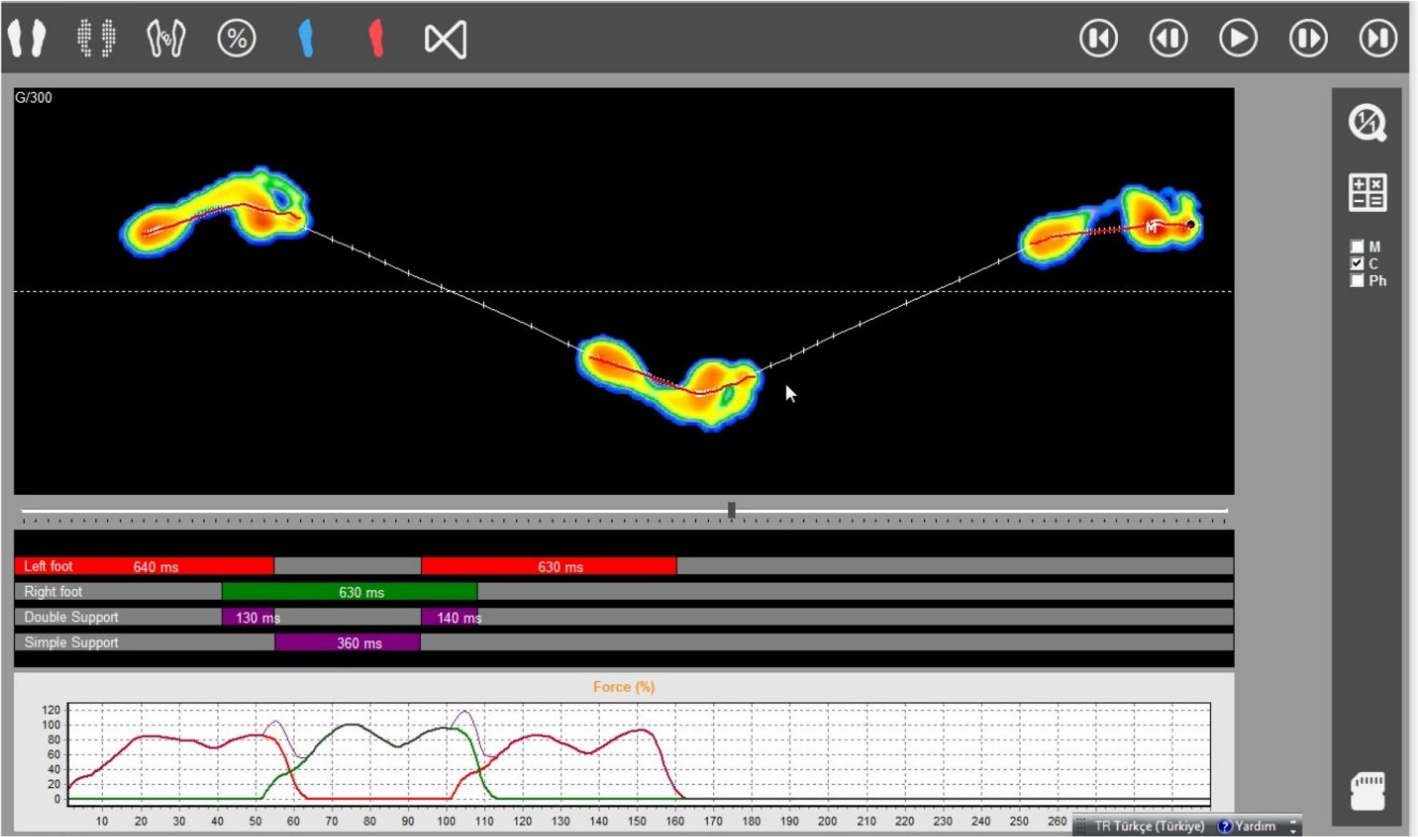
The evaluation of spatiotemporal gait parameters using the Win-Track platform

**Fig. 2:**
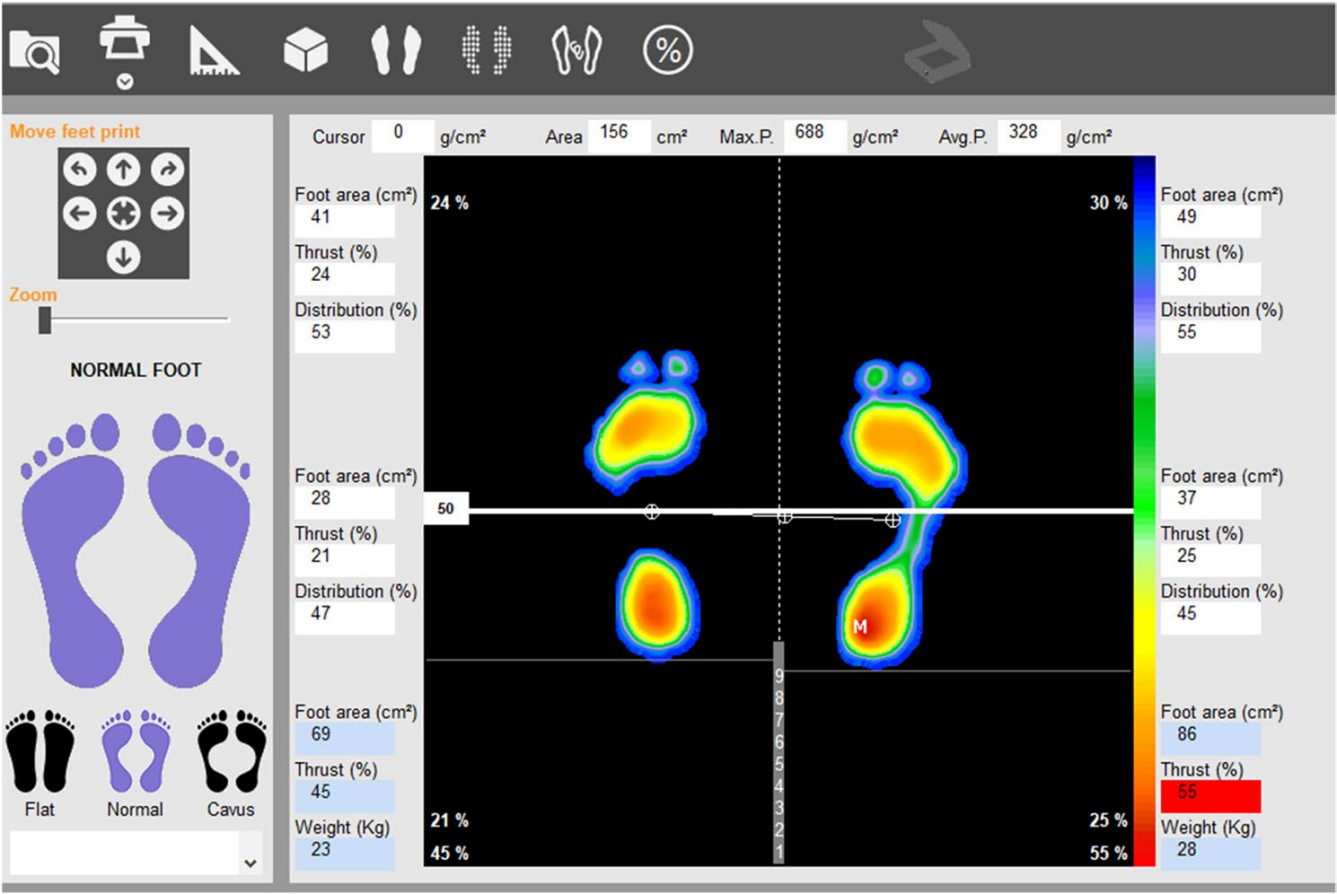
The evaluation of weight transfer in static stance using the Win-Track platform

The Win-Track platform (MEDICAPTEURS Technology, France) is an instrumented system to assess plantar pressures and gait parameters during barefoot walking. It evaluates static posture and dynamic gait characteristics with dimensions of 1610 mm×652 mm×30 mm and a 9 mm thickness. Collected data are transferred to a computer-based interface, automatically identifying footstep patterns and calculating gait parameters, providing clinicians with an objective analysis of walking mechanics. This study employed the three-step protocol, which offers higher test-retest reliability than the one-step protocol (Fig. 1). Participants took three steps beyond the platform, with the heel of the third step marking the starting point. Each participant completed three walking trials. Participants walked at a self-selected pace while looking straight ahead to maintain natural gait patterns. The three-step gait acquisition protocol was used because previous studies evaluating the reliability of the Win-Track platform have shown that this protocol provides higher test–retest reliability for several spatiotemporal gait parameters compared with the one-step protocol [13]. Across the three trials, multiple steps were recorded for each participant, allowing the calculation of spatiotemporal parameters for both the left and right limbs. The mean values obtained from the recorded steps were used for statistical analysis. Recorded spatiotemporal gait parameters included step cycle duration, single-support phase duration, swing phase duration, step length, foot angle, cadence, and gait cycle distance [4]. The foot angle was defined as the angle between the direction of progression and the longitudinal axis of the foot, determined from the footprint as the line connecting the center of the heel to the forefoot region. Step cycle duration was the interval between the initial contacts of opposite feet, while the single-support phase lasted from foot contact to heel-off. The swing phase measured the elevated foot duration. Step length was the distance between consecutive heel contacts, and foot angle was calculated between the longitudinal axis and calcaneus. Cadence reflected steps per minute, whereas gait cycle distance represented the anterior-posterior distance between successive heel contacts [4, 10]. Furthermore, the participants were evaluated based on their weight transfer in static stance, with the evaluation divided into right foot, left foot, forefoot, and hindfoot categories (Fig. 2).

#### Maximum plantar pressure

Maximum plantar pressure is the peak pressure exerted at a specific point in the plantar region during walking. If two steps were recorded on the same lower extremity during the participant’s dynamic gait cycle, the device captures and registers the highest pressure value observed [10].

#### Lequesne index

The Lequesne Index is a disease-specific assessment scale designed to evaluate osteoarthritis severity, comprising three sections: pain or discomfort, maximum walking distance, and activities of daily living. Patients were assessed using the Lequesne Index, with scores recorded on a 24-point scale [14].

#### Oxford knee score

The Oxford Knee Score is a validated and reliable assessment tool designed to evaluate knee-related quality of life and the effectiveness of treatment interventions. It consists of 12 items, each scored on a 5-point scale, ranging from 1 (best outcome) to 5 (severe disability) [15].

#### Pain catastrophizing scales

The scale measures the extent of an individual’s exaggerated negative cognitive and emotional response to pain, commonly called pain catastrophizing. It is a 13-item, 5-point Likert-type scale, with each item scored as follows: 0 (none), 1 (mild), 2 (moderate), 3 (severe), and 4 (always). A higher total score indicates greater pain catastrophizing [16].

#### Western ontario and mcmaster universities osteoarthritis index

The WOMAC is a validated tool for assessing OA, endorsed by Outcome Measures in Rheumatology Clinical Trials. It includes 24 questions across pain (20 points), stiffness (8 points), and physical function (68 points) subscales, where higher scores indicate greater impairment [17].

### Statistical analysis

Statistical analyses were conducted using SPSS 22.0. Since the data were non-normally distributed (Skewness/Kurtosis: -2 to + 2), the Mann-Whitney U test examined gender and BMI differences (*p* < 0.05). Comparisons according to gender were performed as exploratory analyses due to the unequal distribution of male and female participants in the study sample. Correlation and regression analyses were conducted to examine the relationships between spatiotemporal gait parameters and questionnaire-based measures of KOA symptom severity and functional impairment. In addition, the predictive value of spatiotemporal gait parameters was evaluated using multiple linear regression models. Because all participants had bilateral knee osteoarthritis, spatiotemporal gait parameters were analyzed separately for the right and left limbs to account for potential asymmetries related to pain, joint degeneration, and compensatory gait strategies.

## Results

In this study, 25 participants (83.3%) were female and 5 (16.7%) were male. The mean age of the female participants was 60.4 ± 8.96 years, whereas the mean age of the male participants was 61.2 ± 5.21 years (*p* = 0.93), (Table 1). Male participants had a longer gait cycle distance (mm) (female: 895 ± 141.04, male: 1013.80 ± 130.31; *p* = 0.03) and higher left forefoot weight distribution (%) (female: 24.60 ± 8.28, male: 32.20 ± 12.26; *p* = 0.02), (Table 1).

**Table 1:** Demographic, clinical, and gait characteristics of the participants

Variable	Female (*n* = 25)	Male (*n* = 5)	*p*-value	Effect size (*r*)
Age (years)	60.4 ± 8.96	61.2 ± 5.21	0.93	0.02
BMI (kg/m²)	32.16 ± 5.03	29.22 ± 1.67	0.10	0.30
BMI Classification				
Overweight (25–29.9 kg/m²)	13 (43.3%)			
Obese (30–39.9 kg/m²)	17 (56.7%)			
Lequesne Index	8.74 ± 3.73	9.10 ± 5.03	0.94	0.10
Oxford Knee Score	36.00 ± 5.09	35.20 ± 8.04	0.61	0.18
Pain Catastrophizing Scale	11.08 ± 5.87	9.80 ± 7.82	0.68	0.10
WOMAC Pain	7.56 ± 3.79	7.80 ± 3.83	0.78	0.05
WOMAC Joint Stiffness	2.52 ± 1.56	3.00 ± 1.73	0.61	0.18
WOMAC Function	25.64 ± 12.02	25.20 ± 14.25	0.91	0.02
WOMAC Total	35.72 ± 16.91	36.00 ± 19.74	0.95	0.01
Left Forefoot Weight (%)	24.60 ± 8.28	32.20 ± 12.26	0.02*	0.43
Left Heel Weight (%)	26.13 ± 9.83	23.60 ± 11.70	0.50	0.18
Total Weight (%)	51.93 ± 14.27	55.40 ± 24.00	0.45	0.13
Right Forefoot Weight (%)	23.40 ± 10.28	24.00 ± 14.02	0.90	0.03
Right Heel Weight (%)	24.63 ± 10.22	20.60 ± 10.71	0.31	0.17
Total Right Weight (%)	48.03 ± 14.27	44.60 ± 24.00	0.65	0.08
Max. Pressure Left (g/cm²)	1558.97 ± 241.80	1667.00 ± 319.10	0.45	0.13
Max. Pressure Right (g/cm²)	1534.87 ± 238.44	1665.60 ± 266.88	0.49	0.14
Step Duration Left (ms)	636.67 ± 90.98	656.00 ± 83.25	0.62	0.14
Step Duration Right (ms)	648.67 ± 66.84	654.00 ± 62.29	0.76	0.05
Gait Cycle Duration Left (ms)	1271.43 ± 155.93	1383.33 ± 115.47	0.45	0.18
Gait Cycle Duration Right (ms)	1257.31 ± 124.02	1285.00 ± 117.90	0.74	0.10
Single Stance Duration Left (ms)	365.00 ± 43.27	-	-	-
Single Stance Duration Right (ms)	565.71 ± 297.82	460.00 ± 0.00	0.61	0.18
Double Stance Duration Left (ms)	402.33 ± 78.60	463.33 ± 132.79	0.67	0.14
Double Stance Duration Right (ms)	412.00 ± 82.65	485.00 ± 110.91	0.47	0.19
Swing Duration Left (ms)	1476.52 ± 187.28	1530.00 ± 194.16	0.55	0.12
Swing Duration Right (ms)	1468.62 ± 158.92	1508.00 ± 159.28	0.66	0.11
Stride Duration Left (ms)	2099.00 ± 207.13	2286.67 ± 196.30	0.42	0.23
Stride Duration Right (ms)	2081.54 ± 199.41	2130.00 ± 167.13	0.73	0.10
Step Length Left (mm)	456.17 ± 83.14	485.80 ± 101.19	0.55	0.12
Step Length Right (mm)	472.57 ± 76.22	528.20 ± 42.04	0.25	0.25
Gait Cycle Length (mm)	895 ± 141.04	1013.80 ± 130.31	0.03*	0.40
Gait Cycle Length Right (mm)	940.97 ± 118.43	989.60 ± 116.02	0.54	0.15
Angle Left (°)	4.82 ± 2.09	4.60 ± 2.32	0.75	0.05
Angle Right (°)	4.42 ± 2.34	5.01 ± 4.10	0.62	0.14

Data are presented as mean ± standard deviation for each group. *: *p* < 0.05

The mean BMI was 32.16 ± 5.03 kg/m² in female participants and 29.22 ± 1.67 kg/m² in male participants, with no statistically significant difference between the groups (*p* = 0.10). Based on BMI classification, 13 patients (43.3%) were overweight (BMI: 25–29.9 kg/m²) and 17 patients (56.7%) were obese (BMI: 30–39.9 kg/m²), (Table 1). Total double support duration was longer in obese patients (overweight: 360 ± 45.46 ms; obese: 451.76 ± 83.38 ms) (Table 2).

**Table 2:** BMI-based comparison of lequesne index, oxford knee score, pain catastrophizing scale, WOMAC, and spatiotemporal gait parameters

	Variable	Overweight	Obese	Overweight vs. Obese Comparison	All Participants
		Min - Max Mean ± SD	Min - Max Mean ± SD	*p*	Min - Max Mean ± SD
Questionnaires	Lequesne Index	1.5–15 8.85 ± 4.83	3–11.50 8.66 ± 2.97	0.907	1.5–15 8.74 ± 3.73
	Oxford Knee Score	27–47 35.6 ± 6.73	32–44 36.26 ± 3.88	0.756	27–47 36 ± 5.09
	Pain Catastrophizing Scale	2–20 11.4 ± 7.18	3–20 10.86 ± 5.08	0.829	2–20 11.08 ± 5.87
	WOMAC Pain	1–12 7.5 ± 3.92	0–11 7.6 ± 3.83	0.95	0–12 7.56 ± 3.78
	WOMAC Joint Stiffness	0–4 2.2 ± 1.31	0–5 2.73 ± 1.70	0.413	0–5 2.52 ± 1.55
	WOMAC Physical Function	3–41 25 ± 13.46	5–38 26.06 ± 11.42	0.833	3–41 25.64 ± 12.01
	WOMAC Total	5–54 34.7 ± 18.40	5–54 36.40 ± 16.46	0.811	5–54 35.72 ± 16.90
Spatial	Step Length Right (mm)	305–578 480 ± 69.70	320–594 466.88 ± 82.50	0.649	305–594 472.56 ± 76.22
	Step Length Left (mm)	289–601 441 ± 110.87	390–578 467.67 ± 54.57	0.392	289–601 456.16 ± 83.13
	Gait Cycle Length Right (mm)	812–1109 968.15 ± 108.02	758–1109 920.17 ± 124.93	0.279	758–1109 940.96 ± 118.42
	Gait Cycle Length Left (mm)	672–1109 913.84 ± 141.51	664–1148 915.52 ± 150.80	0.975	664–1148 914.8 ± 144.33
Temporal	Step Duration Right (ms)	540–720 646.92 ± 57.79	560–810 650 ± 74.74	0.903	540–810 648.66 ± 66.83
	Step Duration Left (ms)	420–830 640 ± 123.89	540–780 634.11 ± 58.95	0.864	420–830 636.66 ± 90.98
	Gait Cycle Duration Right (ms)	960–1370 1232.72 ± 126.18	1110–1590 1275.33 ± 123.56	0.398	960–1590 1257.30 ± 124.01
	Gait Cycle Duration Left (ms)	920–1550 1338.75 ± 214.10	1110–1490 1230 ± 94.34	0.123	920–1550 1271.43 ± 155.92
	Single Stance Duration Right (ms)	460–1240 620 ± 346.62	430–430 430 ± 0	0.497	430–1240 565.71 ± 297.81
	Single Stance Duration Left (ms)	270–270 270 ± 0	320–400 375.56 ± 29.20	0.009**	270–400 365 ± 43.26
	Swing Duration Right (ms)	1070–1660 1437.50 ± 165.04	1290–1850 1490.58 ± 155.66	0.385	1070–1850 1468.62 ± 158.91
	Swing Duration Left (ms)	1100–1660 1489.16 ± 225.36	1280–1810 1467 ± 162.03	0.766	1100–1810 1476.51 ± 187.27
	Stride Duration Right (ms)	1640–2260 2041.81 ± 183.83	1840–2590 2110.66 ± 211.47	0.395	1640–2590 2081.53 ± 199.41
	Stride Duration Left (ms)	1700–2400 2204.29 ± 263.24	1850–2410 2042.31 ± 152.76	0.096	1700–2410 2099 ± 207.13
	Total Double Support (ms)	260–410 360 ± 45.46	360–620 451.76 ± 83.38	0.001**	260–620 412 ± 82.64
Stability	Angle Right (Degrees)	1.56–9.45 5.22 ± 2.93	1.41–6 3.79 ± 1.59	0.098	1.41–9.45 4.41 ± 2.34
	Angle Left (Degrees)	2.58–9.46 5.31 ± 2.12	1.93–7.70 4.43 ± 2.03	0.267	1.93–9.46 4.82 ± 2.08
	Max Pressure Point Right (g/cm2)	1157–2011 1464.15 ± 257.35	1320–2065 1588.94 ± 214.94	0.159	1157–2065 1534.86 ± 238.43
	Max Pressure Point Left (g/cm2)	1329–1833 1511.84 ± 176.79	1224–2089 1595 ± 281.68	0.360	1224–2089 1158.86 ±
	Right Forefoot Weight (SS) (%)	0–33 23.53 ± 11.26	10–54 23.29 ± 9.81	0.950	0–54 23.4 ± 10.28
	Right Heel Weight (SS) (%)	2–31 20.69 ± 8.62	0–44 27.64 ± 10.54	0.063	0–44 24.63 ± 10.21
	Total Right Weight (SS) (%)	2–58 44.15 ± 18.92	43–72 51 ± 8.87	0.198	2–72 48.03 ± 14.26
	Left Forefoot Weight (SS) (%)	21–54 28.84 ± 10.07	9–46 23.58 ± 8.16	0.125	9–54 25.86 ± 9.26
	Left Heel Weight (SS) (%)	17–48 27.07 ± 10.16	0–39 25.41–9.82	0.654	0–48 26.23 ± 9.83
	Total Left Weight (SS) (%)	42–98 55.76 ± 18.94	28–57 49 ± 8.87	0.203	28–98 51.93 ± 14.26

*BMI* Body max index, *Min* Minimum, *SS* Static standing, *Max* Maximum, *SD* Standard deviation, *mm* Millimeter, *ms* Milliseconds, *g* Gram, *cm* Centimeter, *WOMAC* Western Ontario and McMaster Universities Osteoarthritis Index, *p*: significance *p < 0.05; **p < 0.01

Significant correlations were observed between spatiotemporal gait parameters and osteoarthritis-related assessment scales (Table 3). Step length (right) was negatively correlated with the PCS (*r*=-0.477, *p* = 0.016). Stride duration (right) was positively correlated with the Lequesne Index (*r* = 0.441, *p* = 0.040) and WOMAC Pain (*r* = 0.432, *p* = 0.045). Swing duration (right) was positively correlated with the Lequesne Index (*r* = 0.427, *p* = 0.033) and WOMAC Pain (*r* = 0.413, *p* = 0.040), and negatively correlated with the Oxford Knee Score (*r*=-0.424, *p* = 0.035). Swing duration (left) was positively correlated with the Lequesne Index (*r* = 0.396, *p* = 0.049). Total double support duration was positively correlated with the Lequesne Index (*r* = 0.511, *p* = 0.009) and WOMAC Pain (*r* = 0.448, *p* = 0.025), and negatively correlated with the Oxford Knee Score (*r*=-0.455, *p* = 0.022).

**Table 3:** Correlations between spatiotemporal and stability parameters and scales derived to assess osteoarthritis

		Lequesne Index		Oxford Knee Score		Pain Catastrophizing Scale		WOMAC Pain	
		r	*p*	r	*p*	r	*p*	r	*p*
Spatial	Step Length Right (mm)	-0.097885	0.641583	0.268654	0.194106	-0.477360	0.015819*	-0.192707	0.356061
	Step Length Left (mm)	-0.245579	0.236707	0.241152	0.245533	-0.164887	0.430906	-0.183993	0.378633
	Gait Cycle Length Right (mm)	-0.071966	0.732464	0.179759	0.389892	-0.333532	0.103244	-0.075407	0.720161
	Gait Cycle Length Left (mm)	-0.197276	0.344550	0.248930	0.230167	-0.369006	0.069486	-0.245318	0.237221
Temporal	Swing Duration Right (ms)	0.426639	0.033433*	-0.423732	0.034789*	0.309436	0.132274	0.412873	0.040246*
	Swing Duration Left (ms)	0.396173	0.049935*	-0.305580	0.137417	0.087573	0.677227	0.270635	0.190712
	Stride Duration Right (ms)	0.441362	0.039755*	-0.465523	0.029010*	0.302287	0.171521	0.432214	0.044552*
	Stride Duration Left (ms)	0.264589	0.288679	-0.225578	0.368111	0.035021	0.890274	0.055264	0.827586
	Total Double Support (ms)	0.510638	0.009099**	-0.455595	0.022097*	0.366382	0.071647	0.448222	0.024633*
Stability	Angle Right (Degrees)	0.158271	0.449874	-0.273093	0.186557	0.385191	0.057240	0.075460	0.719971
	Angle Left (Degrees)	0.094197	0.661525	-0.184533	0.388021	0.239815	0.259025	0.128457	0.549706
	Max Pressure Point Right (g/cm2)	-0.183770	0.379222	0.078546	0.708998	-0.179782	0.389829	-0.090267	0.667846
	Max Pressure Point Left (g/cm2)	-0.155030	0.459326	0.071449	0.734318	-0.125419	0.550269	-0.058088	0.782701
	Total Right Weight (SS) (%)	0.075803	0.718749	-0.226746	0.275731	0.156400	0.455320	0.023993	0.909366
	Total Left Weight (SS) (%)	-0.072933	0.728998	0.225476	0.278501	-0.160332	0.443921	-0.021696	0.918011

*mm* Millimeter, *ms* Milliseconds, *g* Gram, *cm* Centimeter, *SS* Static standing, *WOMAC* Western Ontario and McMaster Universities Osteoarthritis Index, *r* pearson’s correlation coefficient, *p* significance *p < 0.05; **p < 0.01

In the final regression model, the gait variables identified in the previous analytical steps were included. Associations between statistically significant spatiotemporal parameters and osteoarthritis questionnaires were examined using regression analysis (Table 4). Regression analysis showed that the Lequesne Index was significantly associated with stride duration (right) (19.5%), swing duration (right) (18.2%), swing duration (left) (15.7%), and total double support duration (26.1%). Regression analysis showed that stride duration (right) was significantly associated with the Oxford Knee Score (21.7%). In addition, swing duration (right) (18%) and total double support duration (20.8%) were also significantly associated with the Oxford Knee Score (Table 4). Step length (right) was significantly associated with PCS scores (22.8%) (Table 4). Stride duration (right) was significantly associated with WOMAC scores (18.7%), while swing duration (right) (17%) and total double support duration (20.1%) also showed significant associations with WOMAC (Table 4). Total double support duration showed the strongest association with the Lequesne Index (36.4%), Oxford Knee Score (43.6%), and WOMAC (36.4%) (*p* < 0.001), whereas step length (right) showed the strongest association with PCS scores (22.8%) (*p* < 0.05) (Table 4).

**Table 4:** The best predictive gait parameters of questionnaires assessing osteoarthritis

DV		Results of Simple Linear			Results of Within Group Stepwise	
	Independent variable	*R*	*r* ^2^	*p*	Δr^2^​​	*p*
Lequesne	Stride Duration Right (ms)	0.441	0.195	0.040*		
	Swing Duration Right (ms)	0.427	0.182	0.033*		
	Swing Duration Left (ms)	0.396	0.157	0.049*		
	Total Double Support (ms)	0.511	0.261	0.009**	0.364	0.003**
Oxford	Stride Duration Right (ms)	0.466	0.217	0.029*		
	Swing Duration Right (ms)	0.424	0.180	0.035*		
	Total Double Support (ms)	0.456	0.208	0.022*	0.436	0.001**
PCS	Step Length Right (mm)	0.477	0.228	0.016*	0.228	0.016*
WOMAC	Stride Duration Right (ms)	0.432	0.187	0.045*		
	Swing Duration Right (ms)	0.413	0.170	0.040*		
	Total Double Support (ms)	0.448	0.201	0.025*	0.364	0.003**

*DV* Dependent variable, *PCS* Pain Catastrophizing Scale, *WOMAC* Western Ontario and McMaster Universities Osteoarthritis Index, *mm* Millimeter, *ms* Miliseconds, *p* significance *p < 0.05. **p < 0.01. In regression analysis only include predictors that are significantly associated with the outcome (see Table 3)

## Discussion

This study examined the relationship between spatiotemporal gait parameters and commonly used OA assessment questionnaires, demonstrating significant associations between gait characteristics and self-reported disease severity, functional impairment, and pain perception. Given that gait analysis devices may not be universally available, these findings contribute to clinical practice by highlighting the relevance of gait parameters in OA evaluation.

### Sex-related differences in gait parameters

The finding that males exhibited longer gait cycles than females is consistent with previous research on sex-related differences in spatiotemporal gait parameters. Previous studies suggest that step length is strongly influenced by height, and when adjusted for height, women may walk slightly faster than men [18]. The longer gait cycles observed in males may therefore reflect anthropometric differences such as height and stride length.

In addition, males demonstrated greater left forefoot weight distribution in the present study. However, forefoot weight distribution was assessed during static standing rather than dynamic gait; therefore, its relationship with walking mechanics should be interpreted with caution. Previous studies in patients with knee osteoarthritis have reported sex-related differences in gait kinematics, with females demonstrating greater knee abduction and hip adduction angles during walking [19]. Furthermore, footprint analysis studies have shown that males tend to have larger plantar contact areas during the stance phase, which may contribute to differences in forefoot loading patterns [20].

### BMI-related differences in gait parameters

In this study, obese individuals demonstrated a longer total double support duration compared with overweight individuals. Prolonged double support time is commonly associated with reduced walking speed, impaired postural stability, and increased joint loading in individuals with knee osteoarthritis. Obesity-related biomechanical changes are known to influence gait patterns by increasing the knee adduction moment and medial knee joint loading [21]. As a result, individuals with higher body mass may adopt a more cautious gait pattern characterized by longer double support phases to improve stability and reduce joint stress. Previous studies have also reported asymmetric gait patterns in patients with knee osteoarthritis, with the affected limb demonstrating longer step times and initial double support [22]. In obese individuals, these adaptations may be more pronounced due to increased biomechanical loading. Furthermore, increased ground reaction forces associated with obesity may contribute to slower gait speed and prolonged double support duration during walking [23, 24].

### Correlations between gait parameters and osteoarthritis-related measures

Even in patients with bilateral knee osteoarthritis, asymmetrical gait adaptations may occur due to differences in pain severity, joint degeneration, or compensatory strategies. Therefore, spatiotemporal parameters were analyzed separately for the right and left limbs.

The present findings demonstrate significant associations between spatiotemporal gait parameters and KOA assessment scales, highlighting the relationship between biomechanical alterations and clinical symptoms. The negative correlation between step length (right) and PCS indicates that higher pain catastrophizing scores are associated with shorter step lengths, consistent with previous studies linking pain catastrophizing to reduced mobility and altered gait patterns in osteoarthritis [22, 25]. In addition, stride duration (right) was positively correlated with the Lequesne Index and WOMAC Pain, suggesting that prolonged stride duration may reflect greater disease severity and pain-related gait adaptations [26, 27]. Similarly, swing duration (right) showed significant associations with the Lequesne Index, Oxford Knee Score, and WOMAC Pain, indicating that alterations in swing phase may be related to pain and functional impairment in KOA [27, 28]. Finally, total double support duration was positively correlated with the Lequesne Index, Oxford Knee Score, and WOMAC Pain, suggesting that prolonged double support time may reflect reduced stability and functional limitations in individuals with knee osteoarthritis [22, 26].

### Association between gait parameters and osteoarthritis severity

The final regression analysis demonstrated significant associations between spatiotemporal gait parameters and the Lequesne Index, a widely used measure of osteoarthritis severity. Longer stride duration, prolonged swing duration (both right and left), and increased double support duration were significantly associated with higher Lequesne Index scores. These findings suggest that individuals with more severe OA exhibit prolonged gait phases, likely reflecting difficulties in weight-bearing and reduced movement stability during walking [29–31].

The prolongation of stride and swing duration observed in this study is consistent with previous research demonstrating gait alterations in individuals with knee osteoarthritis. Patients with KOA often exhibit shorter stride lengths and reduced walking speed compared with healthy individuals, which are commonly attributed to pain, joint stiffness, and functional limitations [31, 32]. Similarly, increased double support duration has been reported as a compensatory strategy adopted to improve stability and reduce joint loading during walking in individuals with more severe disease [33, 34].

From a clinical perspective, these findings highlight the potential value of spatiotemporal gait parameters as objective indicators of osteoarthritis severity. Gait analysis provides quantifiable measures of locomotor alterations that are closely related to clinical outcomes such as pain, functional impairment, and disease progression [35]. Recent studies have also demonstrated that modern gait analysis techniques, including markerless motion capture systems, can reliably assess spatiotemporal gait parameters in patients with OA, further supporting their potential role in monitoring disease progression and guiding rehabilitation strategies [35, 36].

This study also demonstrated significant associations between stride duration, swing duration, and total double support duration and clinical outcomes in KOA, highlighting the predictive value of gait parameters in assessing functional impairment, pain perception, and quality of life. Stride duration (right), swing duration (right), and total double support duration were significant predictors of the Oxford Knee Score, with stride duration explaining 21.7% of its variance. Shorter gait cycle durations and reduced double support times were associated with better knee function, reflecting improved mobility and stability during walking [19, 37].

Step length (right) was significantly associated with PCS scores, explaining 22.8% of the variance, indicating that shorter step lengths are associated with greater pain-related distress [19]. This relationship supports the role of gait adaptations in pain avoidance strategies among individuals with KOA. Furthermore, stride duration, swing duration, and total double support duration showed significant associations with WOMAC scores, suggesting that prolonged gait phases may reflect greater pain-related functional impairment [38]. These findings are consistent with previous research showing that gait slowing and increased double support duration are common compensatory adaptations in individuals experiencing chronic pain and gait instability [19].

The findings of this study highlight a strong relationship between alterations in gait mechanics and pain-related disability in individuals with KOA. Among the examined parameters, total double support duration emerged as the strongest predictor of disability across multiple clinical outcome measures, including the Lequesne Index, Oxford Knee Score, and WOMAC. In addition, step length (right) was identified as a significant predictor of PCS. These findings are consistent with previous studies reporting that individuals with KOA often adopt shorter step lengths as a compensatory strategy to reduce pain and discomfort during walking [39].

Prolonged double support duration may reflect a cautious gait pattern adopted to improve stability and minimize pain during weight transfer. During this phase, both feet remain in contact with the ground, which enhances balance but may reduce overall gait efficiency. Previous research has similarly reported longer stance phases, reduced step length, and slower walking speeds in individuals with osteoarthritis, reflecting compensatory adaptations to pain and joint instability [39, 40].

The strong predictive value of double support duration across multiple clinical outcome measures suggests that this parameter may serve as a useful indicator of disease severity in KOA. Because gait alterations may not always be fully captured by conventional clinical assessments, spatiotemporal gait parameters may provide valuable objective information for evaluating functional impairment and monitoring treatment outcomes in individuals with knee osteoarthritis [41].

### Study limitations

This study has several limitations. The relatively small sample size (*n* = 30) limits the generalizability of the findings, and the gender imbalance in the sample (25 females, 5 males) may affect the applicability of the results to male patients with KOA. Only patients with Kellgren–Lawrence Grade 3 KOA were included, preventing analysis across different disease severity levels. In addition, the cross-sectional design precludes causal inferences, and the use of self-reported questionnaires may introduce response bias. Controlled laboratory conditions may not fully reflect real-world gait patterns, and potentially relevant factors such as muscle strength, proprioception, and psychological variables were not considered. Furthermore, excluding individuals with BMI > 40 kg/m² may limit the applicability of the findings to patients with severe obesity. Additionally, although correlations between spatiotemporal gait parameters and clinical outcomes were identified, the relatively small sample size and imbalance in sex distribution limited the feasibility of multivariable analyses adjusting for potential confounders such as age, sex, and BMI. Although no significant differences in age or BMI were observed within the sample, future studies with larger and more balanced populations are needed to confirm these findings using adjusted statistical models. Although no significant difference in participant height was observed between sexes (*p* > 0.05), leg length was not specifically recorded. Therefore, potential anthropometric influences on gait parameters cannot be completely excluded. The unequal gender distribution may also have limited the statistical power of gender-based comparisons. Future longitudinal studies with larger and more diverse samples are warranted.

### Clinical implications and future directions

The findings of this study have important implications for the clinical management of OA. The substantial predictive value of double support time and step length suggests that gait analysis may be valuable for assessing disease severity and monitoring treatment response. This is particularly relevant in the context of OA, where traditional clinical assessments often fail to capture the full spectrum of functional impairment [41]. Developing interventions to address gait alterations in OA patients is a promising area of research. For example, gait training programs that focus on improving step length and reducing double support time may be beneficial in reducing pain-related disability and improving functional outcomes. Additionally, interventions targeting pain catastrophizing, such as cognitive-behavioral therapy, may help to address the psychological factors contributing to altered gait mechanics [42].

## Conclusion

In conclusion, the findings of this study highlight the complex interplay between gait mechanics, pain-related disability, and psychological factors in OA patients. The substantial predictive value of double support time and step length underscores the importance of gait analysis in assessing disease severity and monitoring treatment response. Further research is needed to elucidate the underlying mechanisms driving these gait alterations and develop effective interventions to improve functional outcomes and reduce disability in OA patients.

## Data Availability

The datasets generated and/or analyzed during the current study are available from the corresponding author upon reasonable request.
